# Structural features determining thermal adaptation of esterases

**DOI:** 10.1093/protein/gzv061

**Published:** 2015-12-07

**Authors:** Filip Kovacic, Agathe Mandrysch, Chetan Poojari, Birgit Strodel, Karl-Erich Jaeger

**Affiliations:** 1 Institute of Molecular Enzyme Technology, Heinrich-Heine-University Duesseldorf, Forschungszentrum Juelich, D-52426 Juelich, Germany; 2 Institute of Complex Systems, ICS-6: Structural Biochemistry, Forschungszentrum Juelich GmbH, D-52426 Juelich, Germany; 3 Department of Physics, Tampere University of Technology, FI-33101 Tampere, Finland; 4 Institute of Theoretical and Computational Chemistry, Heinrich-Heine-University Duesseldorf, D-40225 Düsseldorf, Germany; 5 Institute of Bio- and Geosciences IBG-1: Biotechnology, Forschungszentrum Juelich GmbH, D-52426 Juelich, Germany

**Keywords:** esterase, molecular dynamics, psychrophilic, psychrotrophic, mesophilic, thermophilic bacteria, thermophilicity, thermostability

## Abstract

The adaptation of microorganisms to extreme living temperatures requires the evolution of enzymes with a high catalytic efficiency under these conditions. Such extremophilic enzymes represent valuable tools to study the relationship between protein stability, dynamics and function. Nevertheless, the multiple effects of temperature on the structure and function of enzymes are still poorly understood at the molecular level. Our analysis of four homologous esterases isolated from bacteria living at temperatures ranging from 10°C to 70°C suggested an adaptation route for the modulation of protein thermal properties through the optimization of local flexibility at the protein surface. While the biochemical properties of the recombinant esterases are conserved, their thermal properties have evolved to resemble those of the respective bacterial habitats. Molecular dynamics simulations at temperatures around the optimal temperatures for enzyme catalysis revealed temperature-dependent flexibility of four surface-exposed loops. While the flexibility of some loops increased with raising the temperature and decreased with lowering the temperature, as expected for those loops contributing to the protein stability, other loops showed an increment of flexibility upon lowering and raising the temperature. Preserved flexibility in these regions seems to be important for proper enzyme function. The structural differences of these four loops, distant from the active site, are substantially larger than for the overall protein structure, indicating that amino acid exchanges within these loops occurred more frequently thereby allowing the bacteria to tune atomic interactions for different temperature requirements without interfering with the overall enzyme function.

## Introduction

A steadily increasing demand by the biotechnology industry exists to discover and make available enzymes with high stability and catalytic efficiency at both elevated and low temperatures (de Miguel Bouzas *et al.*, 2006; Joshi and Satyanarayana, 2013). Extremophilic microorganisms living permanently at temperatures above 60°C (thermophiles) or below 20°C (psychrophiles) are a valuable source of such enzymes (Basu and Sen, 2009; Morozkina *et al.*, 2010). As such organisms need to live at thermal equilibrium with their environments, evolutionary pressure resulted in suitable adaptation of all cell components. Although a number of enzymes active at temperatures up to 80°C or below 10°C were isolated from extremophilic microorganisms (Kristjansson, 1989), the molecular mechanisms behind these thermal adaptation processes are still poorly understood. Data collected during the last two decades revealed an array of sequential, structural and physiological factors contributing to the thermal adaptation in an additive and complex fashion. Many case studies analyzed differences between thermophilic and psychrophilic enzymes regarding the number and distribution of salt bridges and hydrophobic interactions, the ratio of polar and hydrophobic accessible surface areas, the number of Pro, Asn, Gly residues as well as the length and amino acid composition of loops (Watanabe *et al.*, 1991; Aguilar *et al.*, 1997; Li *et al.*, 2005; Mandrich *et al.*, 2009; Charbonneau and Beauregard, 2013; Pezzullo *et al.*, 2013). However, a general model explaining protein thermal stability is still not available. Consequently, this limited understanding of structural, biophysical and evolutionary features related to thermostability or thermophilicity of proteins hampers the tuning of enzymes toward these properties. This is emphasized by the fact that random mutagenesis approaches (Asial *et al.*, 2013; Stephens *et al.*, 2014; Tian *et al.*, 2014) still outperform rational protein design approaches in engineering a proteins' thermal properties.

Thermally driven protein dynamics affect catalysis, folding, stability and aggregation through multiple subtle changes of intermolecular interactions (Chelli *et al.*, 2004; Olufsen *et al.*, 2005; Dias *et al.*, 2010). This is not surprising as enzymes often undergo conformational changes during catalysis and therefore may exist as an ensemble of conformers with varying stability and functions (Tokuriki and Tawfik, 2009). The flexibility was sophistically tuned by bacteria during the long evolution time span. Therefore, microorganisms adapted to a certain temperature represent suitable systems to study such orderliness. Mutations in proteins that create a balance between the local active site flexibility and overall rigidity are considered to be adaptive as they promote both enzymatic activity and thermal stability (Kokkinidis *et al.*, 2012). Protein loop regions, unregularly structured elements that are mostly surface exposed and usually the most flexible parts of proteins, are related to the stability and function of many proteins (Henzler-Wildman *et al.*, 2007; Goodey and Benkovic, 2008).

The hormone-sensitive lipase (HSL) family is 1 among 15 presently described lipase families (Arpigny and Jaeger, 1999; Charbonneau and Beauregard, 2013) which all have in common an α/β-hydrolase fold, a Ser-His-Asp catalytic triad and a typical GXSXG-lipase pentapeptide (Ollis *et al.*, 1992; Upton and Buckley, 1995). The family is named after human lipase, which is important for the degradation of triacylglycerol in muscles and is regulated, among other stimuli, by adrenaline (Watt and Spriet, 2004). The tetrapeptide HGGG located close to the N-terminus is a hallmark of HSL family members with the function to stabilize the tetrahedral intermediate. A further characteristic feature of HSL enzymes is the cap-domain covering the active site and composed of several, usually 4–5 α-helices (Mandrich *et al.*, 2009). A number of HSL enzymes lack interfacial activation suggesting that the function of this cap-domain is unrelated to the activation function of the lid-domain observed in some lipases (Verger, 1997). Therefore, the function of the N-terminal part of HSL enzymes (which is part of the cap-domain) was rather linked to substrate specificity and modulation of the catalytic efficiency (Mandrich *et al.*, 2005).

Thermostability is a desirable feature of industrial enzymes which are employed in processes performed at temperatures above 50°C or below 15°C. For example, thermostable lipases are used in lipolytic reactions at high temperatures to increase the solubility and decrease the viscosity of the lipidic substrates (Demirjian *et al.*, 2001). Psychrophilic lipases are valuable additives for energy-efficient processes conducted at low temperatures (Cavicchioli *et al.*, 2002). The spectrum of cold- and hot-adapted lipases encompasses enzymes generally produced by psychrophilic (Joseph *et al.*, 2008; Cai *et al.*, 2009) and thermophilic (van den Burg, 2003) microorganisms. Their biotechnological applications range from food, leather and paper production to use as additives in detergents, and production of biodiesel and pharmaceutical intermediates. Thus, lipases have become almost indispensable for the biotechnology industries (Jaeger *et al.*, 1994, 1999; Jaeger and Eggert, 2002; Panke *et al.*, 2004; Bornscheuer and Kazlauskas, 2006). Among the still existing drawbacks of industrial lipase applications is their missing thermostability making it difficult to withstand industrial reaction conditions.

Here, we have performed comparative biochemical and molecular dynamics (MD) studies using carboxylesterases identified in bacteria living at temperatures ranging from 10°C to 70°C to establish a system for the study of temperature adaptation mechanisms in nature. To this end, we expressed, purified and biochemically characterized EstS, a psychrophilic lipase from *Shewanella halifaxensis* (Zhao *et al.*, 2006), EstP, a psychrotolerant lipase from *Pseudomonas* sp.*B11-1* (Choo *et al.*, 1998), EstB, a mesophilic lipase from *Burkholderia thailandensis* (Brett *et al.*, 1998) and Est2, a thermophilic lipase from *Alicyclobacillus acidocaldarius* (Darland and Brock, 1971). These α/β-hydrolases with 38–47% of sequence identity emerged to be suitable from an evolutionary, biochemical and structural perspective for the study of natural adaptation processes. Indeed, their thermal properties resemble the temperatures of their bacterial host habitats and structural and MD studies revealed flexible surface-exposed loop regions as the most relevant parts for tuning their thermal properties. Hence, these esterases represent a convenient system to study the structure–function relationships determining protein thermal properties both experimentally and by MD simulations.

## Materials and methods

### Database search and sequence alignment

The amino acid sequences were analyzed and aligned using the Blast search and alignment tool of the Universal Protein Knowledge Base (www.uniprot.org) (Magrane and Consortium, 2011). The three-dimensional (3D) structure of Est2 (De Simone *et al.*, 2000) was retrieved from the Protein Data Bank (PDB, www.rcsb.org) (Berman *et al.*, 2000).

### Comparative modeling and validation

The Modeller 9v8 (Eswar *et al.*, 2006) software was used for homology modeling of EstS, EstP and EstB using the crystal structure of Est2 (De Simone *et al.*, 2000) (PDB ID: 1EVQ) as a template. The structures obtained from homology modeling may involve steric clashes and suboptimal geometries, hence it is required to successfully minimize the structures before continuing with the MD simulations. To this end, the models were processed with the *pdb2gmx* GROMACS tool to generate the molecular topology and then centered in a box with dimensions of 8.5 × 8.5 × 8.5 nm^3^. For resolving possible steric clashes, the models were initially energy-minimized in vacuum using the steepest descent method together with the GROMOS96 53A6 force field as implemented in GROMACS 4.5.4 (Oostenbrink *et al.*, 2004). Minimization was performed until a negative potential energy was reached and the maximum force on each atom was <1000.0 kJ mol^−1^ nm^−1^. The minimized models were solvated using SPC (Berendsen *et al.*, 1981) water molecules and counter ions were added to balance the enzyme charge. The solvated models were also minimized using the same criteria as in vacuum in order to relax the possible clashes between protein and solvent. Failure to minimize the structures would lead to instabilities during the subsequent MD simulations. After minimization, the C_α_ atoms of the different models were superimposed on the template structure and for each esterase, i.e. EstS, EstP and EstB, the model with least root-mean-square deviation (RMSD) was selected for further studies. The stereochemical parameters of the homology models were evaluated using the PROCHECK v.3.5.4 program (Laskowski *et al.*, 1993). Additionally, the models were validated using qualitative model energy analysis (QMEAN) (Benkert *et al.*, 2008) and protein structure analysis (PROSA) (Wiederstein and Sippl, 2007) programs. The protein structures were analyzed, aligned and visualized using PyMol (DeLano Scientific LLC) and SwissPDB viewer (Guex and Peitsch, 1997). Protein secondary structure assignment was performed with DSSP (Kabsch and Sander, 1983). The atomic interactions were calculated using the Protein Interactions Calculator tool with default settings (Tina *et al.*, 2007).

### MD simulations

All MD simulations were carried out using the GROMACS 4.5.4 simulation package (Hess *et al.*, 2008) using, as for the minimization the GROMOS96 53A6 force field (Oostenbrink *et al.*, 2004) and SPC water (Berendsen *et al.*, 1981) as solvent. The enzymes were simulated at temperatures ranging from 15°C to 90°C to explore the molecular mechanisms of cold and hot adaptations (Supplementary Table SI). An initial equilibration under isothermal–isochoric (NVT) conditions was performed for 250 ps during which the protein heavy atoms were restrained with a force constant of 1000 kJ mol^−1^ nm^−2^. Here, the v-rescale thermostat (Bussi *et al.*, 2007) with a coupling constant of 0.1 ps was used to regulate the temperature of the enzyme and solvent/ions separately. The systems were then equilibrated under isothermal–isobaric (NPT) conditions for 1 ns. For the NPT ensemble, isotropic Parrinello–Rahman pressure coupling (Parrinello and Rahman, 1981) was used with a time constant of 2.0 ps maintaining a constant pressure of 1 bar. An isothermal compressibility of 4.5 × 10^5^ bar^−1^ was applied in all box dimensions. Long-range electrostatic calculations were performed using the Particle Mesh-Ewald method (Darden *et al.*, 1993; Essmann *et al.*, 1995) in connection with periodic boundary conditions. Van der Waals and Coulombic interaction cut-offs were set to 1.2 nm, and the LINCS algorithm (Hess *et al.*, 1997) was used to constrain all bond lengths. Following equilibration, production MD runs were performed for 100 ns. Here the parameter settings were similar to the NPT equilibration step, except that all restraints were removed. The use of virtual sites for hydrogens allowed us to use time step of 5 fs for integration with coordinates and velocities saved every 20 ps for analysis. All analyses were performed using GROMACS analysis tools. The backbone RMSD and C_α_ root-mean-square fluctuations (RMSF) were calculated for the last 30 ns of each simulation trajectory as all simulations reached equilibration within 70 ns (Supplementary Fig. S1). At the end of simulations, all structures were intact, i.e. no unfolding had occurred. Protein secondary structure assignment was performed with DSSP (Kabsch and Sander, 1983).

### Expression and purification of esterases

Genes *estS*, *estB* and *est2* were amplified by standard PCR using *Pfu* DNA polymerase (Invitrogen) and the genomic DNA of *S. halifaxensis*, *B. thailandensis* and *A. acidocaldarius* as templates, respectively (Supplementary Table SII). The genomic DNA was purified with the aid of DNeasy tissue kit (Qiagen). Specific primers (Supplementary Table SII) encoding restriction sites for *Nde*I and *Xho*I for *estS* and *estB* were used. All primers were synthesized by MWG Biotech (Ebersberg, Germany). The *EstP* gene with *Nde*I and *Xho*I restriction sites was synthesized by GeneArt AG (Regensburg, Germany) due to unavailability of genomic DNA of *Pseudomonas* sp. strain B11-1. All genes were cloned in the pET28a vector with *Nde*I and *Xho*I or *Sac*I restriction sites yielding pET28-*EstS*_His6_, pET28-*EstP*_His6_, pET28-*EstB*_His6_ and pET28-*Est2*_His6_ plasmids (Supplementary Table SII) for a bacteriophage T7-RNA polymerase-dependent expression from the T7 promoter.

For the expression of lipases, *Escherichia coli* BL21(DE3) was transformed with expression plasmids pET28-*estS*_His6_, pET28-*estP*_His6_, pET28-*estB*_His6_ and pET28-*est2*_His6_. Cultures were grown initially for 2 h at 37°C following 48 h at 15°C or 37°C in an autoinduction medium (Li *et al.*, 2011) containing yeast extract (2.4%, w/v), tryptone (1.2%, w/v), glycerol (0.5%, w/v), lactose (2.0%, w/v) and glucose (0.4%, w/v) in KP_i_ buffer (100 mM, pH 7). The cell density was monitored at 580 nm. After expression, the cells were harvested by centrifugation for 30 min at 5000*g* and 4°C (Sorvall RC 5B Plus, SLC 4000, Thermo Scientific), resuspended in Tris–HCl buffer (20 mm, pH 8.0), and disrupted by two sonication cycles of 3 min each (50%, 20 watt Branson-Sonifier W250) with the samples cooled on ice. The esterases with His_6_-tag fused on their C-terminal end were purified from the soluble cell fraction obtained after centrifugation for 30 min at 16 000*g* and 4°C by affinity chromatography on Ni-NTA agarose (Qiagen) as described previously (Lescic Asler *et al.*, 2010). The proteins were analyzed by polyacrylamide gel electrophoresis under denaturation conditions (SDS–PAGE) on 12% (w/v) gels as described by Laemmli (1970). The protein concentration was determined by the method of Bradford with bovine serum albumin as a standard (Bradford, 1976).

### Biochemical analysis of lipases

#### Substrate specificity

Lipase activities toward fatty acid esters of *p*-nitrophenol (*p*-NP) were determined according to the method of Manco *et al.* (1998). The enzymatic reactions were performed in a 96-well microplate by adding 5 µl of enzyme sample to 200 µl of substrate.

#### Temperature optimum

Enzymatic activities were measured over a range of *p*-nitrophenyl butyrate concentrations from 0.2 to 1.4 mM and temperatures from 5°C to 90°C. Assays were performed in a 96-well microplate by adding 2 µl of enzyme sample to 200 µl of substrate. Kinetic parameters, *K*_m_ and *k*_cat_, were calculated from three independent experiments, where the data were fitted to the Michaelis–Menten equation using a non-linear regression method.

#### Thermostability

The stability of lipases was measured every 20 min during 3 h incubation at temperatures ranging from 5°C to 80°C with *p*-nitrophenyl butyrate (0.2 mM). After incubation, lipase activities were measured at optimal enzyme temperature. Assays were performed in a 96-well microplate by adding 2 µl of enzyme sample to 200 µl of substrate.

## Results

### Selection of target esterases

We have investigated the structure–function relationship of four homologous enzymes originating from psychrophilic, psychrotrophic, mesophilic and thermophilic bacteria to study enzyme adaptation to extreme temperatures. As model enzymes, we selected lipases from the α/β-hydrolase family as they are ubiquitous enzymes with a large number of 3D structural data available. In the first step, a model lipase with known 3D structure, biochemical function and enzyme kinetic parameters was selected. Out of all bacterial ester hydrolases (EC 3.1) with an α/β-hydrolase fold available in the PDB, the following enzymes were excluded: (a) enzymes from pathogenic organisms, (b) membrane-bound enzymes, (c) enzymes with low-resolution structures (>3 Å), (d) enzyme structures without primary literature data being available, (e) heteromultimeric enzymes and (f) enzymes with structures solved by methods other than X-ray crystallography. This approach revealed 17 esterases (Supplementary Table SIII), whose sequences were subjected to a Blast search to identify bacterial homologs belonging to three different groups of temperature optima. Only homologous enzymes fulfilling criteria (a)–(f) were selected and had to pass as further requirements: (g) sequence identity between 35 and 50% to the lipase with known structure, (h) sequence length <400 amino acids, and (i) conserved catalytic triad residues. The growth temperatures of the bacterial hosts retrieved from the literature were used to classify the enzymes as psychrophilic, psychrotrophic, mesophilic and thermophilic.

This approach revealed three model lipases, one from the thermophilic bacterium *A. acidocaldarius* and two from the mesophilic bacteria *Bacillus amyloliquefaciens* and *Burkholderia glumae* (Supplementary Table SIII). Among them, esterase Est2 from *A. acidocaldarius* (De Simone *et al.*, 2000) is structurally, kinetically and mutationally well characterized, can be easily expressed and was thus chosen for our thermal adaptation studies. From a pool of nine Est2 homologues, the esterases from *S. halifaxensis* (EstS), *Pseudomonas* sp.*B11-1* (EstP) (Choo *et al.*, 1998) and *B. thailandensis* (EstB) were selected as psychrophilic, psychrotrophic and mesophilic enzymes for our studies (Supplementary Table SIV). Here, factors like sequence identity, availability of literature data and availability of bacterial strains for molecular biology work were considered during the selection process.

### Bioinformatic analysis

**Fig. 1:**
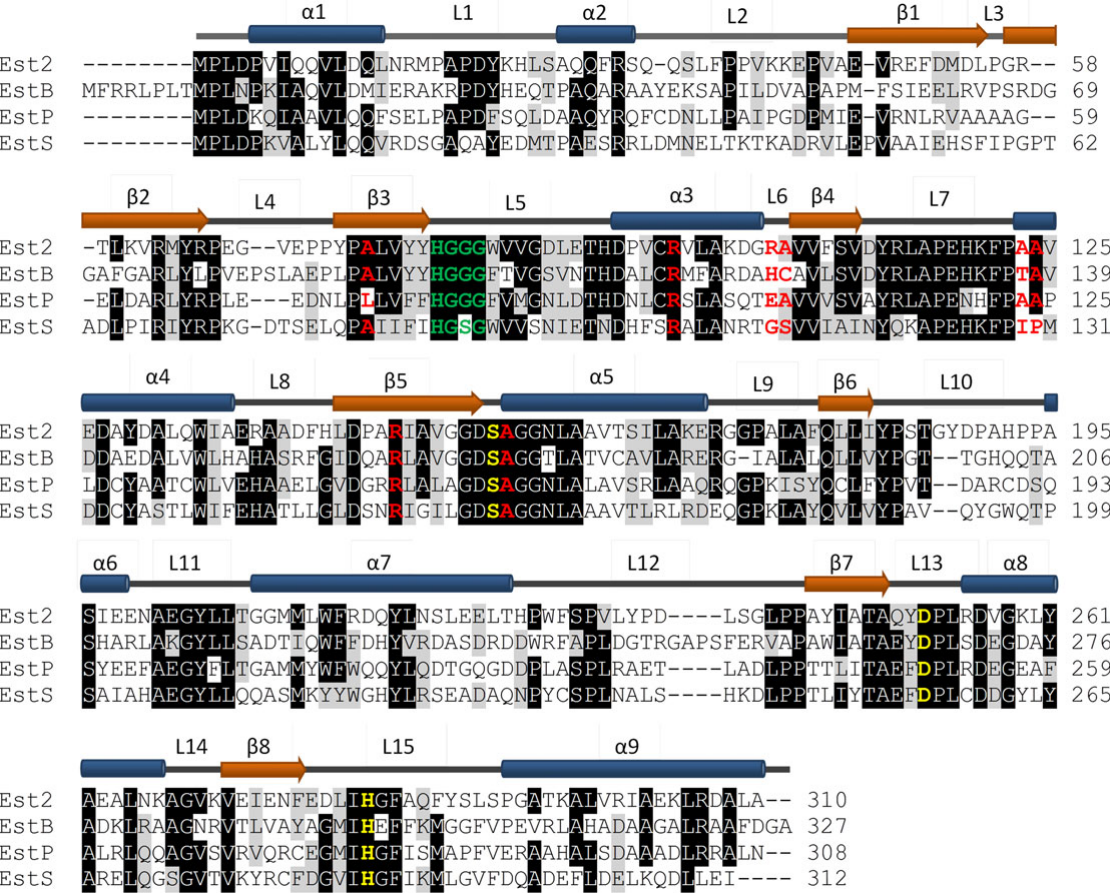
Structure-based sequence alignment of esterase Est2 from the thermophilic bacterium *A. acidocaldarius* with esterases EstB from mesophilic *B. hailandensis*, EstP from psychrotrophic *Pseudomonas* sp. B11-1, and EstS from psychrophilic *S. halifaxensis*. Notably, loops show lower sequence similarity when compared with the other regions. Sequences identical and similar in at least three residues were shaded in black and gray, respectively. The catalytic triad residues Ser, Asp and His are colored in yellow and the oxyanion hole residues in green. Residues colored in red follow the traffic rule reported for Est2 (Mandrich *et al.*, 2009). Secondary structural elements of Est2 are shown above the alignment with α-helices as blue cylinders, β-strands as red arrows, and loops as gray lines, respectively (De Simone *et al.*, 2000).

The sequence alignment of the four chosen esterases revealed strictly conserved active site residues, including the catalytic serine embedded in the GXSXG sequence typical for lipases, catalytic His and Asp residues and the typical oxyanion hole HGGG-motif located in front of the catalytic serine residue (Fig. 1). These properties classify the esterases as members of the HSL subfamily. Analysis of the amino acid content of enzymes from this subfamily revealed that a decrease in Cys, Asn, Thr content and an increase in Glu and Pro content correlate with an increasing optimal growth temperature of the corresponding host bacteria (Manco *et al.*, 1998). Among the chosen esterases, these tendencies were confirmed for Cys, Thr, Pro and, in addition, revealed an increasing content of Val with increasing thermophily (Supplementary Fig. S2 and Table SV). Slight deviations from these trends were observed for Asn and Glu in mesophilic EstB. Asn, whose side chain undergoes deamination at elevated temperatures and thus contributes to the thermal instability of enzymes (Robinson, 2002), is underrepresented in the thermophilic Est2 (6 residues) compared with cold lipases (9 and 8 Asn residues in EstS and EstP, respectively).

From the analysis of mesophilic and thermophilic enzymes, a ‘traffic rule’ for the HSL family was postulated. The changes Gln → Arg, Gly → Arg, Thr → Arg and Ser → Ala, where the first amino acid is the one found in the mesophilic and the second one in the thermophilic enzyme, were identified as statistically significant (Argos *et al.*, 1979; Mandrich *et al.*, 2004). However, from the eight changes detected in the *Bfae*–Est2 pair (mesophilic–thermophilic pair), none was observed in the group of enzymes studied in this work (Fig. 1), questioning the regularities derived from a small set of protein structures or sequences.

### Enzyme purification and characterization

In contrast to the previously reported esterase activity for Est2 (Manco *et al.*, 1998) and EstP (Choo *et al.*, 1998), the functions of EstS and EstB were predicted only by sequence homology to known lipases. The three Est2 homologs studied here show a sequence identity of ∼45%. The respective genes were cloned, expressed, the enzymes purified and assayed for substrate specificity. Similar biochemical properties of the four enzymes represent a prerequisite for comparison of their thermal properties. Upon expression at 37°C, enzymatically active Est2, EstB and EstP were obtained, but EstS did not show enzymatic activity (Supplementary Fig. S3). We succeeded to produce enzymatically active EstS by lowering the expression temperature to 15°C. Moreover, we observed that a temperature of 15°C was also favorable for the expression of EstB and EstP, resulting in 30 and 50% more enzymatic activity, respectively, when compared with expression at 37°C (Supplementary Fig. S3B).

**Fig. 2:**
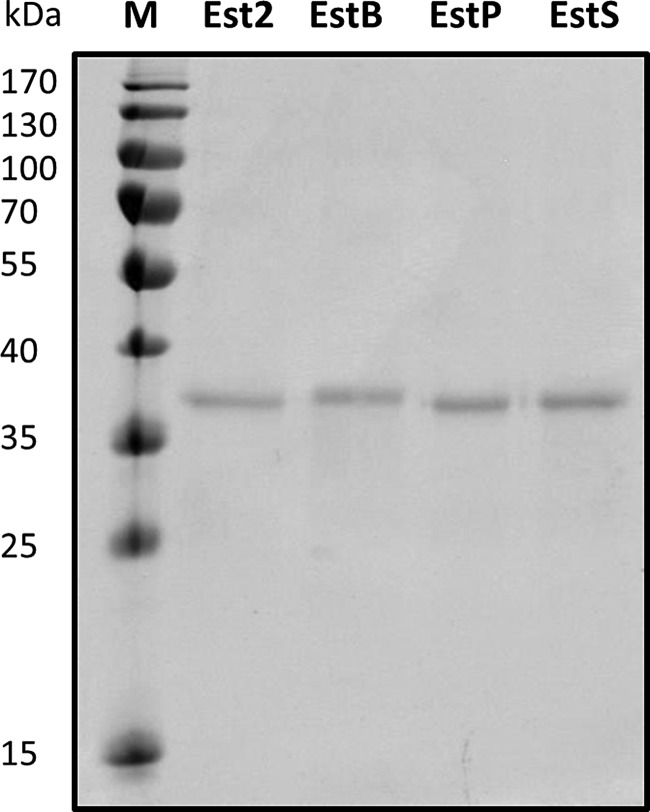
SDS–PAGE analysis of purified recombinant esterases Est2, EstB, EstP and EstS. Enzymes carrying a His_6_-tag at their C-terminal end were purified by the IMAC method, separated by SDS–PAGE on a 12% (w/v) polyacrylamide gel and stained with Coomassie Brilliant Blue G-250. Molecular weights of standard proteins are indicated on the left.

All four enzymes were purified to electrophoretic homogeneity using metal affinity chromatography (Fig. 2 and Supplementary Fig. S4) and their specific activities were measured using *p*-nitrophenyl esters with fatty acids of chain lengths from 2 to 18 carbon atoms (Table I) by the method described for Est2 (Manco *et al.*, 1998). None of the proteins showed activity toward esters of long-chain fatty acids with 16 or 18 carbon atoms (data not shown). Our data are similar to previously reported substrate specificity of Est2 (Manco *et al.*, 1998), indicating a negligible effect of the His-tag on the catalytic properties of Est2. All enzymes had a preference for short-chain fatty acids with 4 or 6 carbon atoms. EstS and EstB showed the highest activity for the C4-ester, EstP as well as Est2 for the C6-ester. It is notable that EstB hydrolyzed a broader spectrum of *p*-nitrophenyl esters (from C2 to C12) than EstP and EstS which did not hydrolyze C10- and C12-esters. However, comparison of the substrate specificities of Est2, EstB, EstP and EstS clearly revealed that these four esterases represent biochemically similar enzymes, thus providing a solid basis for comparison of their thermal properties.

**Table I:** Substrate specificities of Est2, EstB, EstP and EstS using as substrates *p*-nitrophenyl esters of fatty acids with 2, 4, 6, 8, 10, 12 and 14 carbon atoms, respectively

Substrate	Specific activities (U mg^−1^)^a^			
	Est2	EstB	EstP	EstS
*p*-nitrophenyl acetate	744.9	1.3	12.9	22.7
*p*-nitrophenyl butyrate	1321.7	19.5	22.8	42.8
*p*-nitrophenyl hexanoate	1838.5	11.9	29.3	9.5
*p*-nitrophenyl octanoate	886.7	10.0	1.0	n.d.
*p*-nitrophenyl decanoate	337.0	2.8	n.d.	n.d.
*p*-nitrophenyl dodecanoate	84.4	2.2	n.d.	n.d.
*p*-nitrophenyl myristate	18.3	n.d.	n.d.	n.d.

^a^The reaction was carried out at the temperature optima of the enzymes, at 70°C (Est2), 40°C (EstB), 35°C (EstP) and 30°C (EstS) under standard conditions. All values represent means of at least three independent measurements with SDs of <10%.

### Thermostability and thermophilicity

**Fig. 3:**
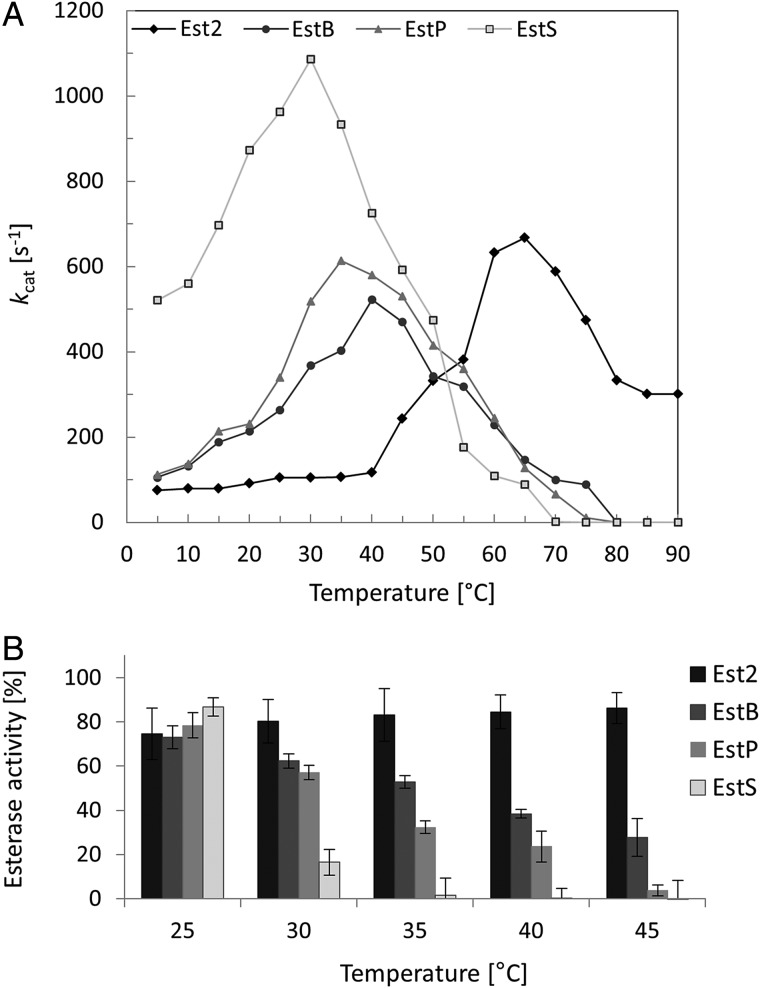
Effect of temperature on activity and stability of esterases Est2, EstB, EstP and EstS. (**A**) The temperature optima were determined with *p*-nitrophenyl butyrate as the substrate at temperatures ranging from 5°C to 80°C. For comparison, Est2 values were divided by 10. Data are mean values of at least three independent measurements with standard deviations <10%. (**B**) The thermal stability of esterases Est2, EstB, EstP and EstS was determined after 1 h incubation at 5–80°C followed by measuring the residual activities with *p*-nitrophenyl butyrate as the substrate at 30°C. Esterase activity is expressed as percentage of the activity of samples before incubation. Data are mean values of at least three independent measurements with bars indicating the standard deviations.

The thermal properties of EstS, EstP, EstB and Est2 originating from bacteria with optimal growth temperatures at 10°C, 20°C, 37°C and 70°C, respectively, were analyzed by determination of enzyme activities at temperatures from 5°C to 90°C using the method described for Est2 (Manco *et al.*, 1998) with *p*-nitrophenylbutyrate instead of *p*-nitrophenylbutyrate as the substrate. Kinetics measurements revealed optimal temperatures of 30°C, 35°C, 40°C and 65°C for EstS, EstP, EstB and Est2, respectively (Fig. 3A).

Temperature stability measurements for the four esterases during incubation for up to 3 h at temperatures from 5°C to 80°C revealed high stability for Est2 in the range of 30–75°C where it retained more than 80% of its activity after 1 h incubation. In contrast, temperatures below 30°C caused inactivation of Est2; e.g. 1 h incubation at 10°C resulted in 48% activity loss (Fig. 3B and Supplementary Table SVI). EstB retained activity after 1 h incubation at temperatures above 35°C when compared with EstP and EstS; under the same conditions, EstP retained more activity than EstS (Fig. 3B). After 1 h incubation of EstB, EstP and EstS at their optimal temperatures, 38% (at 40°C), 29% (at 35°C) and 18% (at 30°C) of residual activity were preserved, respectively. On the other hand, EstS was more stable at higher temperatures than EstP and EstB at low temperature of 5°C, indicating its psychrophilic nature.

### Homology modeling and MD simulations

The different thermal properties of the four esterases were further analyzed by modeling their 3D structures and subsequent subjection to a comparative MD analysis. Est2, a thermophilic homolog with sequence identity above 37% to the other three esterases, represents a suitable template for successful homology modeling as a cut-off of 30% sequence identity is commonly applied (Xiang, 2006). The validation of the geometry of the predicted models with PROCHECK revealed that at least 98% of the residues were in favored regions and <2.2% in outer regions. This is comparable with 0.8% of residues found in the outer regions for the structure of Est2, indicating a good quality of the EstS, EstP and EstB models (Supplementary Fig. S5 and Table SVII). The validation of the overall model quality with PROSA, which is based on the so-called *Z*-score and measures the deviation of the total energy of the structure with respect to an energy distribution derived from random conformations, revealed that the structural models lie well within the range of scores of protein structures that were solved by either X-ray crystallography or NMR (Supplementary Fig. S6 and Table SVII). Furthermore, validation of the homology models by QMEAN analysis revealed *Z*-scores for EstS, EstP and EstB of 0.76, 0.82 and 0.77, respectively. This parameter is calculated using major geometrical parameters of protein structures (torsion angels, secondary structures, solvent accessibility of residues) and can take on values between −4 and 4. However, for structures solved by X-ray crystallography, it is only in the range from 0 to 1. In conclusion, the validation parameters indicate highly reliable homology models for all three proteins, which are suitable for subsequent structural comparison analysis.

**Fig. 4:**
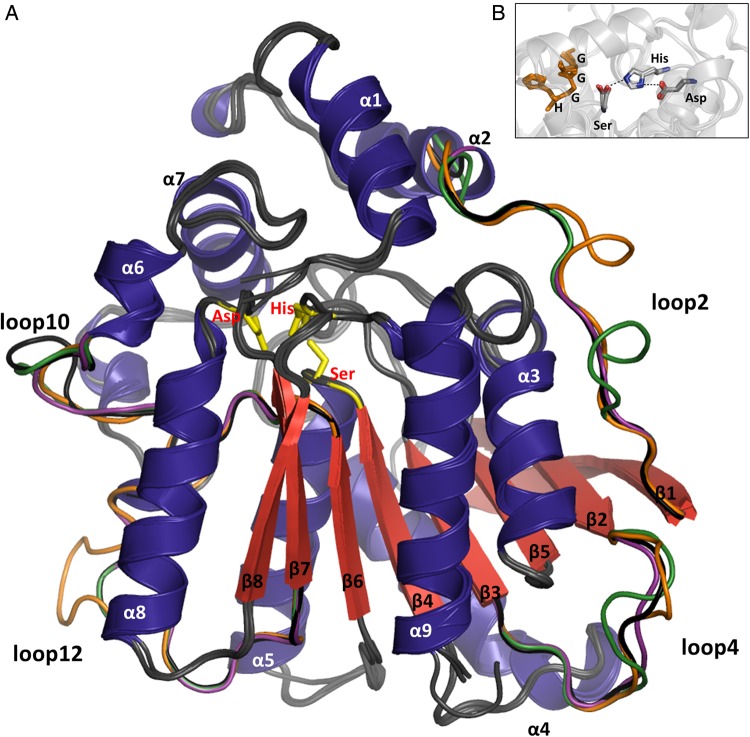
Structural comparison of esterases Est2, EstB, EstP and EstS. (**A**) Superimposition of the structure of Est2 (PDB ID: 1EVQ) with homology models of EstB, EstP and EstS. Secondary structural elements are labeled according to the structure of Est2. Loops with significant structural differences between the four structures are colored in black (Est2), orange (EstB), magenta (EstP) and green (EstS). For simplicity, structurally conserved loops are colored in gray and eight N-terminal amino acids of EstB that are not conserved among this group of enzymes are omitted. The catalytic residues Ser155, Asp252 and His282 of Est2 are shown as stick models in yellow. (**B**) Superimposition of the active sites of Est2, EstB, EstP und EstS. Orientations and distances of the catalytic triad residues are appropriate for establishing the H-bond network important for the catalytic activity of these esterases. Distances between catalytic Ser and His of Est2, EstB, EstP and EstS are 2.7, 3.8, 3.5 and 2.8 Å, respectively, and between His and Asp 3.2, 3.0, 3.1 and 2.7 Å. The HGGG motif containing two Gly residues forming the oxyanion hole close to the catalytic serine are indicated in orange. The catalytic triad residues are colored by element with carbon in gray, nitrogen in blue and oxygen in red; dotted lines represent H-bonds.

The superposition of the EstS, EstP and EstB homology models with the structure of Est2 revealed low RMSD values (<0.44 Å) (Table II) and high conservation of secondary structure elements (>90%) as expected for proteins with similar sequence and function. No significant differences were observed in the main folds of the four enzymes, despite their different thermal properties. The structures of EstS, EstB and EstP resemble the α/β-hydrolase fold with an eight-stranded central β-sheet surrounded by five α-helices and another four α-helices (α1, α2, α6 and α7) organized into the cap-domain covering the active site (Fig. 4A). The active site Ser residue is placed at the top of a sharp *γ*-turn and is at H-bond distance from His, which in turn interacts via an H-bond with Asp, the third residue of the catalytic triad (Fig. 4B). The HGGG-motif close to the N-terminus, which involves two Gly residues that together with Asp located in front of the catalytic Ser help to stabilize the tetrahedral intermediate, is strongly conserved among all four structures. The analysis of number and nature of atomic interactions in all four lipases revealed a significant decrease in the total number of interactions with lower temperature optimum, i.e. in the direction of the psychrophilic lipase EstS (Table II). In total, Est2 (824) showed more interactions than EstB (782) and EstP (784) and these enzymes showed more interactions than EstS (749). It is notable that Est2 had twice as many ion–ion interactions than EstS, what is in agreement with the hypothesis that ion–ion interactions are particularly important for the thermal stabilization of HSL enzymes (Pezzullo *et al.*, 2013). To test this hypothesis, we analyzed the conservation of 24 ion bridges detected in Est2. We found that only a small portion (5 in EstS, 11 in EstP and 8 in EstB) of these ion bridges were conserved (Supplementary Table SVIII). The analysis of the 3D distribution of the salt bridges revealed a similar distribution of salt bridges at the protein surface for all four esterases (Table III). Interestingly, two experimentally confirmed surface-exposed thermostabilizing ion bridges (D145-R148, R31-E118) (Pezzullo *et al.*, 2013) and the ion–ion network formed by E50, K61, R63, E91 and D94 present in Est2 are conserved in the other three esterases under study (Supplementary Table SVIII). The third experimentally confirmed thermostabilizing salt bridge of Est2 (E43-K102) is conserved in mesophilic EstB, but not in EstP and EstS. It should be noted that several salt bridges found in EstS and EstP are not structurally conserved in Est2 (Supplementary Table SVIII). These results support the hypothesis that the ion bridges are generally important for the thermal stability of these enzymes independent of the temperature stability range. However, they also indicate that protein stabilization is achieved by the optimization of the 3D arrangement of ion–ion interactions in proteins rather than just the number of ion–ion interactions.

**Table II:** Structural comparison of EstS, EstP, EstB and Est2

Enzyme	RMSD^a^ (Å)				% SSE^b^	Residues (%)		
	Total	α-Helix	β-Strand	Loops		α-Helix	β-Strand	Loops
EstS	0.40 (302)	0.34 (104)	0.27 (57)	0.42 (130)	90	35	18	46
EstB	0.39 (302)	0.30 (104)	0.29 (55)	0.51 (128)	95	33	17	50
EstP	0.40 (304)	0.27 (105)	0.22 (57)	0,41 (132)	95	36	19	45
Est2	–	–	–	–	–	35	19	46

^a^Number of superimposed C_α_ atoms are given in parentheses.

^b^Percentage of residues assigned to the same secondary structure element as in Est2.

**Table III:** Comparison of interactions of EstS, EstP, EstB and Est2

Interactions	Est2	EstB	EstP	EstS
Hydrophobic	196	183	194	180
Aromatic	18	15	17	16
H-bonds	587	563	560	541
Ion–ion^a^	24 (42/58)	21 (38/69)	16 (62/71)	12 (49/70)
Total	824	782	784	749

^a^Values in parentheses indicate percentage of ion–ion interactions in loop regions and at the protein surface (loops/surface exposed).

The experimental analysis of Asn residues susceptible to thermal deamidation in 23 different proteins with known 3D structures revealed a higher probability of deamination if Asn is followed by a Gly, Ser, His, Ala or Asp residue (Robinson and Robinson, 2001). Analysis of our group of esterases revealed the presence of such pairs in psychrophilic EstS (N99-D100, N238-A239) and in psychroptrophic EstP (N119-H120) (Fig. 1). Interestingly, in Est2, these three Asn residues are substituted by His, Leu and His, respectively. These results suggest that stabilization of thermophilic enzymes from the HSL family is gained by the mutation of potentially unstable Asn residues.

Loops, a structural element related to different aspects of protein function, folding and dynamics, represent almost 50% of the esterase structures under study (46% for EstS, 45% for EstP, 50% for EstB and 46% for Est2) (Table II). Moreover, the most pronounced structural differences between the four esterases were observed in the loop regions. The RMSD values for the loops are higher (0.41–0.51 Å) than for the α-helices and β-strands (0.22–0.34 Å) (Table II). This correlates with the observation that loop regions show less sequence similarity compared with α-helices and β-strands (Fig. 1). Among the 15 loops in the four proteins, the differences between surface-exposed loops 2, 4, 10 and 12 are more pronounced than for other loops (Figs 1 and 4). This is underlined by the finding that the average RMSD for these four loops is higher (EstP: 0.59 Å, EstS: 0.71 Å, EstB: 0.77 Å) than for all loops in the proteins (0.41–0.51 Å). In addition, the sequence similarity of these four loops is lower (31–42%) than for all loops in general (48–60%).

**Fig. 5:**
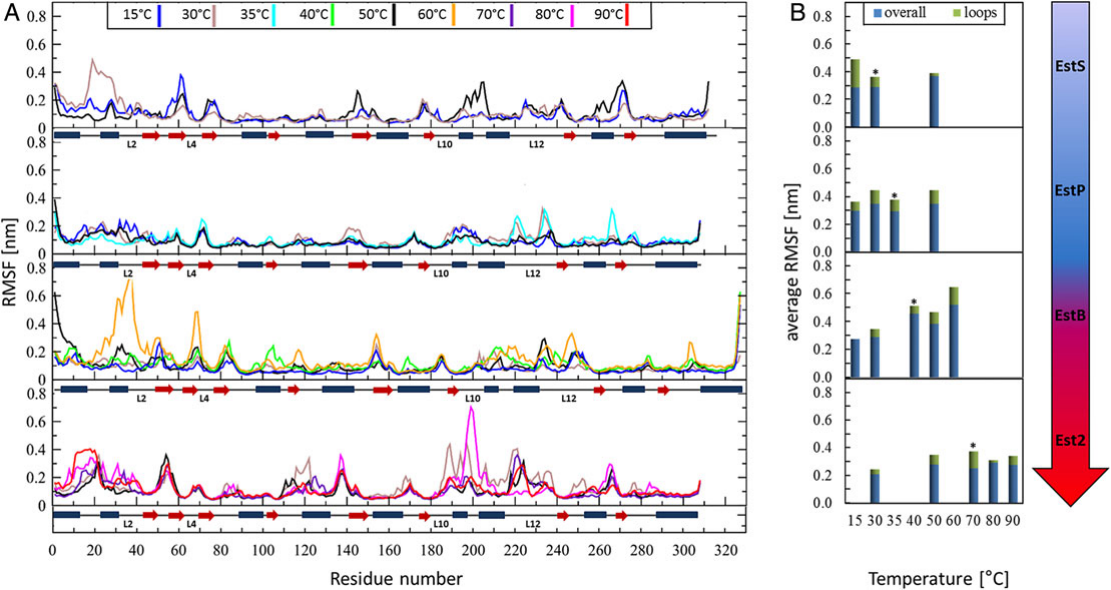
MD simulations of EstS, EstP, EstB and Est2 movements at various temperatures. (**A**) MD simulations were performed for 100 ns and RMSF values for each residue were calculated for the last 30 ns of each simulation trajectory based on the fluctuations of the C_α_ atoms. Secondary structure elements, α-helices (blue rectangles) and β-strands (red arrows), are displayed along the sequences; flexible loops are labeled L2, L4, L10 and L12, respectively. (**B**) Comparison of RMSF values for overall enzyme structures and loop regions. Average RMSF of overall structures are represented by blue bars, and the further increment of the RMSF values for all loops in the proteins are indicated by green bars. Optimal temperatures for each protein are indicated by an asterisk above the bar. In A and B, results are shown for different esterases as indicated by the arrow on the right with blue color indicating optimal activity at lower and red at higher temperatures.

**Fig. 6:**
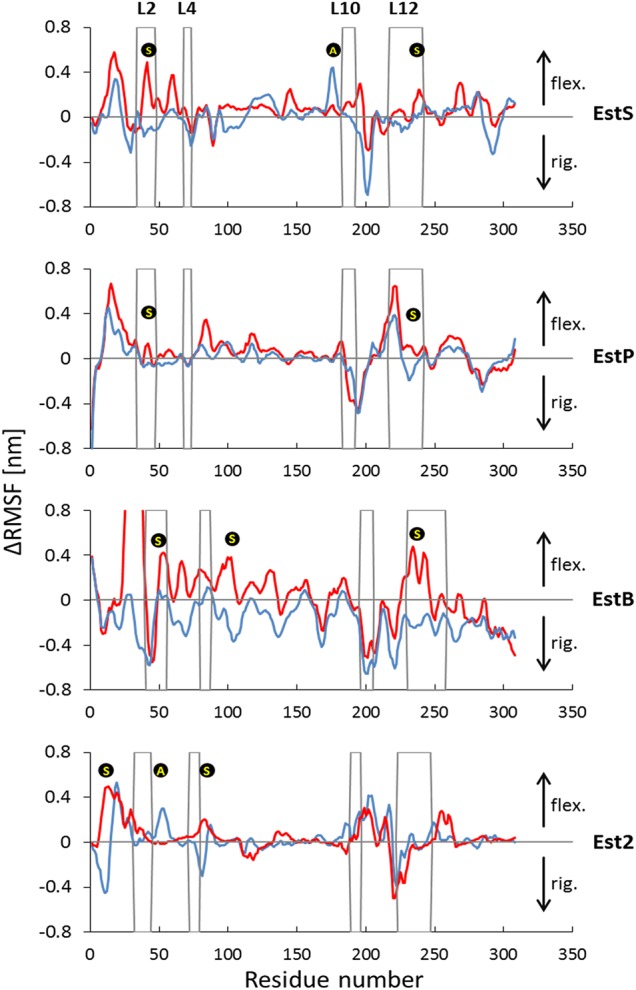
Temperature-induced fluctuations of EstS, EstP, EstB and Est2. The red lines of the fluctuation plots indicate changes in RMSF between optimal temperature for enzyme catalysis (*T*_opt_) and higher temperatures, *T*_high_, for EstS, EstP, EstB and Est2 were 50°C, 50°C, 60°C and 80°C, respectively. The blue lines of the fluctuation plots indicate changes in RMSF between optimal temperature for enzyme catalysis (*T*_opt_) and lower temperatures, *T*_low_, for EstS, EstP, EstB and Est2 were 15°C, 15°C, 15°C and 50°C, respectively. Gray frames indicate the position of loops L2, L4, L10 and L12. Regions proposed to be related to protein stabilization and activity are indicated by 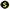 and 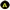, respectively. Arrows at the right-hand side indicate an increase in inflexibility or rigidity.

Many remarkable biological functions in a protein and their profound dynamic mechanisms can be revealed by simulating their internal motions (Khan *et al.*, 2015), and studying their dynamic processes (Gramany *et al.*, 2015). Therefore, further insights into the structural integrity of these esterases with different thermal properties were provided by MD simulations at physiologically relevant temperatures ranging from 15°C to 90°C. These revealed slightly lower RMSF values for Est2 compared with its mesophilic and cold-adapted homologs (Fig. 5) which are in agreement with biochemical analysis. The loop regions represent the most flexible parts in all four esterases. Furthermore, it seemed that the flexibility of the loops is optimized for the optimal temperatures for catalysis. The ratio of the average RMSF for all residues compared with the residues in loop regions at optimal temperatures is ∼0.75 for EstS, EstP and Est2, and, with 0.85, slightly higher for EstB. The four surface-exposed loops (L2, L4, L10 and L12), previously identified as the structurally most dissimilar parts among the four esterases, exhibited a similar flexibility as the other loops at the optimal catalysis temperatures (Fig. 5B). However, these loops became more flexible at temperatures higher (observed for all esterases) than the optimal temperature (Fig. 6). On the opposite, at temperatures lower than the optimal temperatures, these loops were more rigid (Fig. 6). It is notable that these four loops themselves are very flexible (particularly loops 2 and 12) or surrounded by flexible regions (loops 2, 4 and 10) (Figs 5A and 6). These results indicate that the dynamics of the loop regions is generally important for the function of the four esterases and that the most flexible loops are relevant for the stability of the proteins.

## Discussion

Enzyme thermostability and thermophilicity are important for biological function, molecular evolution and biotechnological applications (Asial *et al.*, 2013). Although many lessons have already been learned about protein thermal properties from *in vitro* (Jochens *et al.*, 2011; Wijma *et al.*, 2014) and *in vivo* (Razvi and Scholtz, 2006; Liszka *et al.*, 2012; Asial *et al.*, 2013) experiments, the molecular adaptation related to these properties is still poorly understood. During millions of years, enzymes from extremophile microorganisms evolved to be catalytically efficient at different environmental conditions. For this reason, bioprospecting for these microorganisms represents one of the most promising approaches for the discovery of extremozymes (Liszka *et al.*, 2012; Tchigvintsev *et al.*, 2014; Prasad *et al.*, 2014). Here, we used extremozymes aiming to understand how they naturally adapted to different temperatures. Several case studies and comparative analyses of homologous proteins from organisms of two (Pack and Yoo, 2005; Ratcliff *et al.*, 2009; Mereghetti *et al.*, 2010; Vemparala *et al.*, 2011) or three (Svingor *et al.*, 2001; Bell *et al.*, 2002; Bae and Phillips, 2004) different temperature groups revealed a variety of parameters all affecting thermostability, and conformational flexibility was identified as a key parameter relevant for the balance between thermostability and thermophilicity. However, the molecular and structural mechanisms of cold and hot adaptations still have to be elucidated.

Here, we present a comparative study of natural enzyme adaptation to temperatures between 10°C and 70°C. We have focused on α/β-hydrolases, enzymes from one of the largest and functionally diverse family with probably the oldest fold architecture (Ollis *et al.*, 1992; Upton and Buckley, 1995; Winstanley *et al.*, 2005). An important requirement was structural similarity with yet sufficient evolutionary distance (<50% sequence identity) to ensure that a natural adaptation process driven by the temperature of the respective habitats had occurred (Rost, 1997). On the other hand, we have limited the evolutionary distance (>30% sequence identity) to minimize effects of genetic drift (Arnold, 2009). As a result of applying rigorous sequence, structure, biochemical, biological and environmental criteria, thermophilic (Est2), mesophilic (EstB), psychrotrophic (EstP) and psychrophilic (EstS) esterases from a HSL subfamily were selected for our studies (Supplementary Table SIV).

The enzymes studied here, namely EstS, EstP, EstB and Est2, represent biochemically similar enzymes (Table I), although slight differences in their substrate specificities indicate possible structural differences of the fatty acid binding pocket in the active site. Thirty-six N-terminal residues comprising α-helices α1 and α2, and loop L1 of Est2 were reported to be important for the substrate specificity of Est2 (Mandrich *et al.*, 2005). These regions in particular are highly conserved in sequence (Fig. 1) and structure (Fig. 4) as would be expected for enzymes with similar substrate specificity. Our studies showed that thermostability (Supplementary Table SVI) and temperature optima (Fig. 3) increased going from psychrophilic to thermophilic esterases, in line with the hypothesis of adaptive evolution of protein thermal properties (Fields, 2001; D'Amico *et al.*, 2003; Arnold, 2009). Furthermore, the typical thermophilic properties that we observed for Est2 are in agreement with those already reported for this enzyme (Manco *et al.*, 1998). EstS originating from a psychrophilic bacterium shows thermal properties (high stability at low temperatures, low stability at high temperatures and low optimal temperatures for catalysis) characteristic for psychrophilic enzymes. EstB has a rather mesophilic character, while EstP exhibited characteristics between those of mesophilic and psychrophilic enzymes.

The increase in catalytic activities of cold-adapted enzymes when compared with mesophilic and thermophilic enzymes may be predominantly ascribed to an increase in flexibility (Olufsen *et al.*, 2005). The thermostability of proteins adapted to high temperatures could be primarily attributed to the higher frequency of residues found in a well-packed state than in an extended state (Pack and Yoo, 2005). It is generally accepted that the thermolability of naturally evolved cold enzymes at higher temperatures mostly results from the weakening of all types of interactions; the same is true for thermophilic enzymes at low temperatures (Goldstein, 2007). Our analysis of EstS, EstP, EstB and Est2 confirmed that several of these general rules for cold and warm adapted proteins also apply to these four esterases. Similar to previously described lipases from the HSL subfamily and other enzymes, they adapt to higher temperatures by creating more interactions (Table III), which stabilize the structures at higher temperatures (Mandrich *et al.*, 2009). Also, the increasing abundance of prolines, known to reduce the flexibility of the polypeptide main chain (Saelensminde *et al.*, 2009), and decreasing abundance of asparagines, known to undergo deamination at high temperatures (Robinson and Robinson, 2001; Robinson, 2002), in the direction of thermophilic Est2 were observed (Supplementary Fig. S2). Such global analyses are usually inconclusive with respect to the fact that thermal properties are not determined by just a single factor but rather through the additive combination of many small effects (Pezzullo *et al.*, 2013).

Indicatively, many protein features (ion bridges, residue packing, flexible regions, solvent–residue interactions) found to affect thermal properties can be assigned to the protein surface (Fields, 2001; Li *et al*., 2005). Therefore, we propose an adaptation route for the modulation of protein thermal properties through the optimization of local flexibility at the protein surface. To this end, we have analyzed the correlation of structural differences at the protein surface and temperature-induced alterations of the surface flexibility using four thermally adapted esterases. Our approach is challenged by several facts. First, on average, only a small number (3–4%) of all residues are crucial to achieve correct folding implying that, in principle, almost 96% of the residues are feasible candidates for mutations toward the desired thermal properties (Rost, 1997). Secondly, Rost has shown that the sequence identity for most of the close structural homologs is below 9% (Rost, 1997), making classic analysis based on the amino-acid type ineffective in comparison to atom interaction-based approaches. Obviously, conservation of atomic interactions is unambiguous to preserve protein structure and function (Todd *et al.*, 2001; Glasner *et al.*, 2006). Thirdly, the fast and the slow dynamics of proteins are linked to both thermostability and thermophilicity (Henzler-Wildman *et al.*, 2007). This leads to the assumption that a combination of atomic interactions and protein motions in a complex way determines thermal properties.

As expected, no significant differences were observed in the main fold (Fig. 4) of the four esterases, despite their different thermal properties (Fig. 3). Presumably, other structural features are related to their thermal properties. The contribution of several ion bridges at the surface of Est2 (Pezzullo *et al.*, 2013), which was also found in other proteins such as, for example, *E.coli* phytase (Fei *et al.*, 2013) and ribosomal protein L30e (Chan *et al.*, 2011), was proposed to be essential for their increased thermal stability. However, the contribution of salt bridges appears to be relatively moderate for many other proteins (Goldstein, 2007). The analysis of ion–ion interactions in the four esterases revealed indeed an increase in ion bridges in Est2 compared with its colder homologs (Table III). However, the four ion bridges structurally conserved among the esterases under study were confirmed by mutations to be responsible for the thermostability of Est2 (Supplementary Table SVIII). Thus, we cannot undoubtedly denote ion–ion interactions as a thermostabilizing feature of HSLs without performing further mutagenesis studies.

The most striking differences in sequence (Fig. 1) and structure (Fig. 4 and Table II) were found for the surface-exposed loop structures of the four esterases. We have therefore addressed the question whether these differences might be related to the thermal properties of the enzymes. Owing to several properties of loop structures (e.g. surface accessibility, conformational freedom, high frequency of mutations and low structural conservation), they were suggested as elements in protein structures, which could be efficiently used for the thermal adaptation of proteins (Thompson and Eisenberg, 1999; Hermoso *et al.*, 2009). For instance, in thermostable aminotransferase of *Pyrococcus horikoshii* shorter and less flexible loops contribute to its stability at high temperatures through a decrease in the side chain conformational entropy (Matsui and Harata, 2007). Our MD simulations at the optimal temperatures for enzyme activity confirmed that the loop regions are more flexible than the overall protein structures and correlates with fewer interactions found in loop regions. Notably, structures adjacent to these flexible loops also show higher fluctuations compared with overall structures (Fig. 6). This indicates that the dynamics of these loops might possibly affect both local and long-range interactions, which were reported to be important for stability and function of proteins in general (Pezzullo *et al.*, 2013). The comprehensive analysis of MD simulations at temperatures higher and lower than the optimal temperatures for enzyme activity revealed temperature-dependent flexibility of the four loops L2, L4, L10 and L12. These four loops are structurally least conserved between the four studied esterases and, additionally, they are located distant from the active site (Fig. 4). Loop2 and neighboring regions and loop12 behave similarly in the four enzymes (apart from loop 12 in Est2). They fluctuate more upon increasing the temperature and less when lowering the temperature (Fig. 6). Such response to the temperature is expected for structural elements related to thermostability as an increase in flexibility often leads to destabilization of a protein structure (Li *et al*., 2005). Therefore, our analysis indicates the importance of L2 and L12 for thermostability of HSL esterases. The dynamics of the N-terminal region of Est2 differs from that of the other three esterases. This region of Est2 is flexible at higher temperatures and it is rigidified at lower temperatures. Hence, information for thermostability of HSL esterases may be encoded in their N-terminal part. Furthermore, some regions of the four studied esterases respond more strongly to temperature changes. Respectively, these structures might provide variability of interatomic interaction what could be related to protein stability. Interestingly, some regions linked to the loops (L10 of EstS and L2 of Est2) show a significant increase in dynamics upon lowering the temperature, while these regions are not much affected by an increase in the temperature. Increased flexibility is a feature of psychrophilic enzymes by which they preserve catalytic activities at low temperatures (Svingor *et al.*, 2001; Olufsen *et al.*, 2005). Based on the fact that the flexibility of enzymes is necessary for their catalytic activity, we propose the structural elements L10 and neighboring residues in EstS and L2 in Est2 to be important for the activity of these esterases at low temperatures.

In summary, we have analyzed both experimentally and theoretically four enzymes whose thermal properties correspond to the optimal growth temperatures of their bacterial hosts. Our experimental system relies on a comprehensive bioinformatics-based selection of esterases with different thermostabilities and thermophilicities as the most critical step. The experimental validation of the selected system involves protein expression, purification and activity based-thermal analysis of these enzymes. Finally, we performed a comparative analysis to link thermal properties with structural and dynamic features of the enzymes. Such a comparative four-enzyme system thus represents a promising basis for elucidating how bacterial enzymes adapt to different environmental temperatures.

## Funding

This work was supported by a research grant from the German Research Foundation (DFG) within the International Research Training Group 1628 ‘Selectivity in Chemo- and Biocatalysis’ (SeleCa). C.P. and B.S. gratefully acknowledge the computing time granted on the supercomputer JUROPA at Jülich Supercomputing Centre (JSC). Funding to pay the Open Access publication charges for this article was provided by Heinrich-Heine-University Düsseldorf, Germany.

## Supplementary Material

Supplementary Data
