# Supplementary material for: Structural features determining thermal adaptation of esterases
Source: Protein Eng Des Sel. 2015 Dec 7;29(2):65–76. doi: 10.1093/protein/gzv061 (PMC5943684; doi:10.1093/protein/gzv061)
Supplement: Supplementary Data [file gzv061_Supplementary_Data.zip › gzv061supp.docx]

**Legends to supplementary figures**

**Figure S1:** The evolution of the C_α_ root mean square deviations (RMSDs) from the starting structure of the corresponding simulation are shown for all simulations performed in this study. The different simulation temperatures are represented by different colors for each of the four enzymes. The RMSD graph for EstB at 60 °C shows that this simulation needed almost 70 ns for equilibration while all other systems equilibrated within 40 ns.

**Figure S2**: Correlation of amino acid content and temperatures of bacterial habitats. A tendency of increasing Pro, Val and Glu content and decreasing Thr, Asn and Cys content in the direction of thermophilic esterase Est2 was observed. The mole percentage for each residue type corresponds to the number of the residue type in question per 100 residues. R-squared values were calculated for linear regression.

**Figure S3**: Influence of growth temperature on the production of recombinant Est2, EstB, EstP and EstS. A**)** SDS-PAGE analysis of lysates obtained from *E. coli* BL21 (DE3) cells harboring the empty vector (EV, pET28b), or pET28-*EstS*_His6_ (EstS), pET28-*EstP*_His6_ (EstP), pET28-*EstB*_His6_ (EstB) and pET28-*Est2*_His6_ (EstS) expression plasmids. Gene expression was performed at 15°C (a) and 37°C (b) under autoinduction conditions*.* Polyacrylamide gel (12% (*w/v*)) was stained with Coomassie Brilliant Blue G-250. Target proteins, indicated by arrows, were identified according to estimated mass from SDS-PAGE compared to estimated mass from the amino acid sequence. **B)** Esterase activities were assayed with *p*-nitrophenyl butyrate as the substrate with lysates of *E. coli* BL21 (DE3) cells harboring the empty vector (EV, pET28b), or pET28-*EstS*_His6_ (EstS), pET28-*EstP*_His6_ (EstP), pET28-*EstB*_His6_ (EstB) and pET28-*Est2*_His6_ (EstS). Gene expression was performed at 15°C (a) and 37°C (b) under autoinduction conditions*.* The released *p*-nitrophenolate was measured at 410nm and activity was normalized on the cell amount measured as optical density at 580nm. Data are mean values of at least three independent measurements with bars indicating the standard deviation.

**Figure S4**: Western blot analysis of recombinant esterases Est2, EstB, EstP and EstS using HRP conjugated anti-His-tag antibodies. The molecular weights of standard proteins are indicated on the left.

**Figure S5:** Structure validation of LipS (A), Est2 (B), LipP (C) and LipB (D) models using Ramachandran plot analyses; red, brown, and yellow regions represent core, allowed, and generously allowed regions. Residues indicated in red belong to generously allowed and disallowed regions.

**Figure S6:** Validation of structural models for LipS (circle), LipB (square), LipP (triangle) models using PROSA analysis. The Z-scores of all models are within the range of scores calculated for proteins of similar size with experimentally determined structures indicating good overall quality of the built models. Est2 is indicated as a cross. Z-scores of protein chains in PDB determined by X- ray crystallography or NMR-spectroscopy are indicated in light and dark grey respectively.

**Supplementary figures**

**
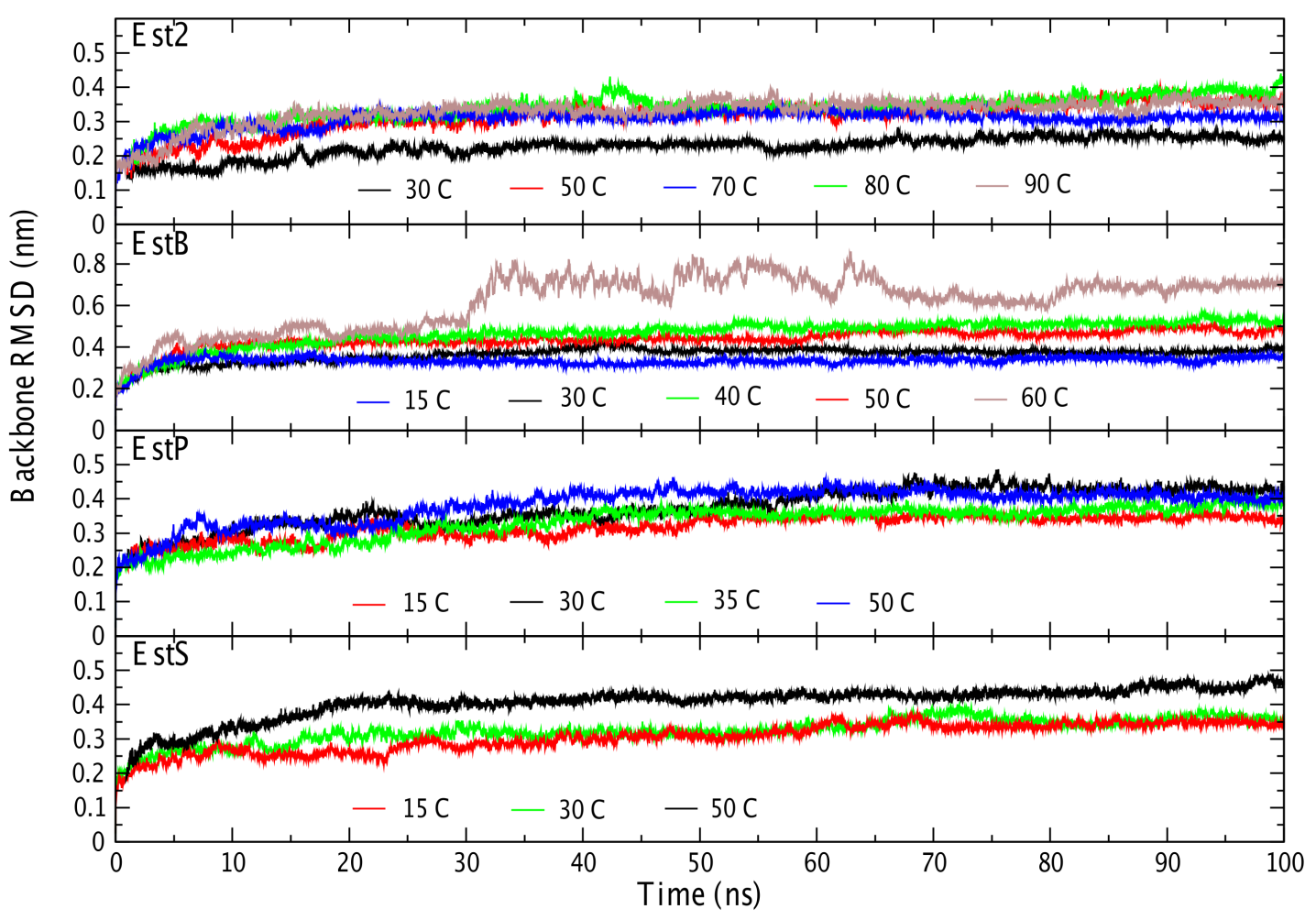
**

**Figure S1**

**
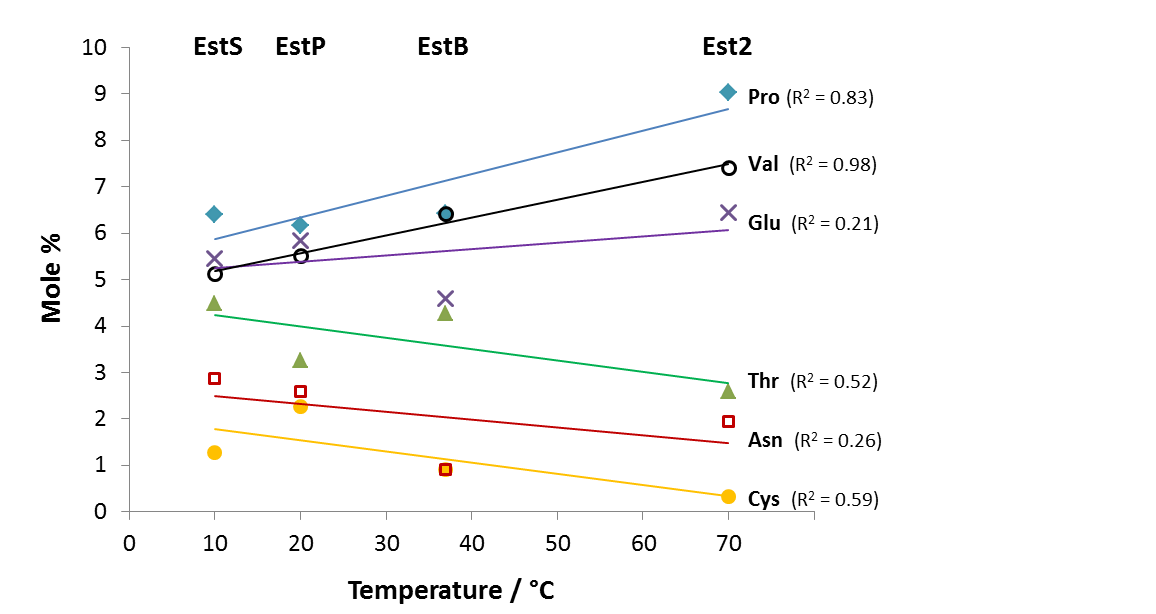
**

**Figure S2**

**
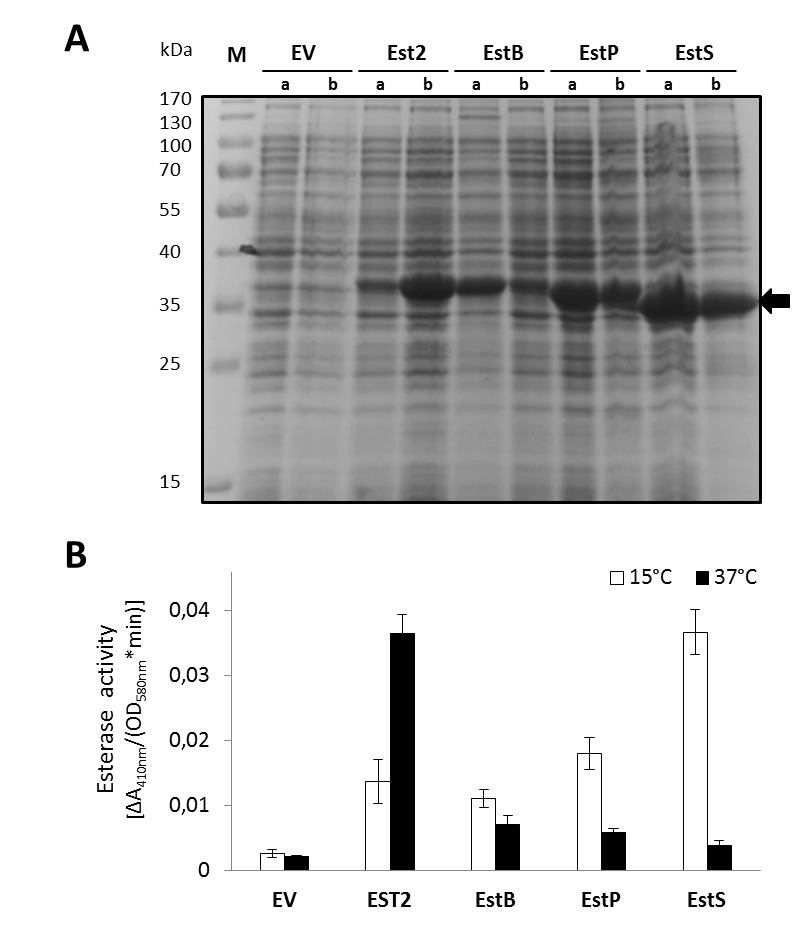
**

**Figure S3**

**
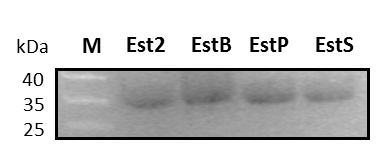
**

**Figure S4**

**
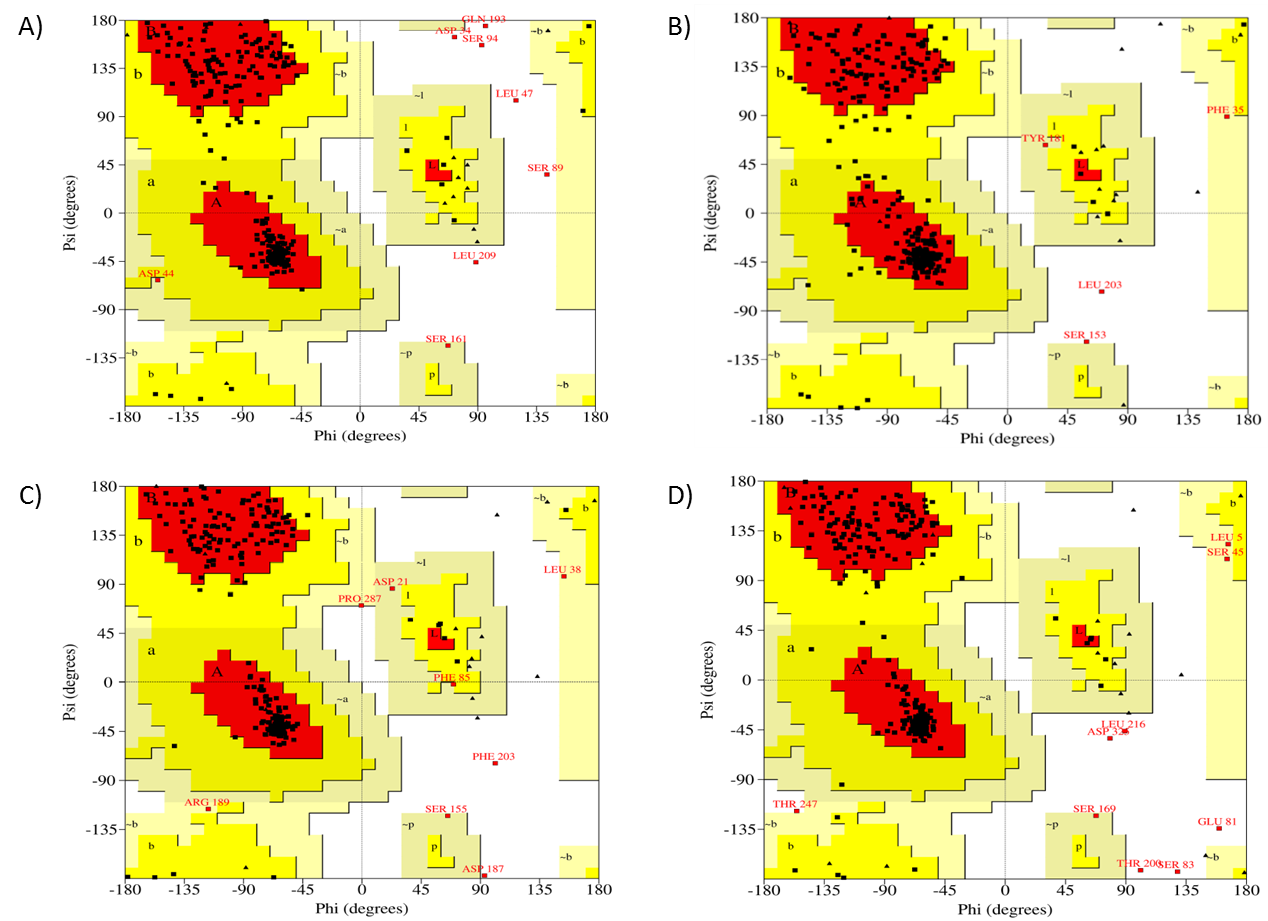
**

**Figure S5**

**
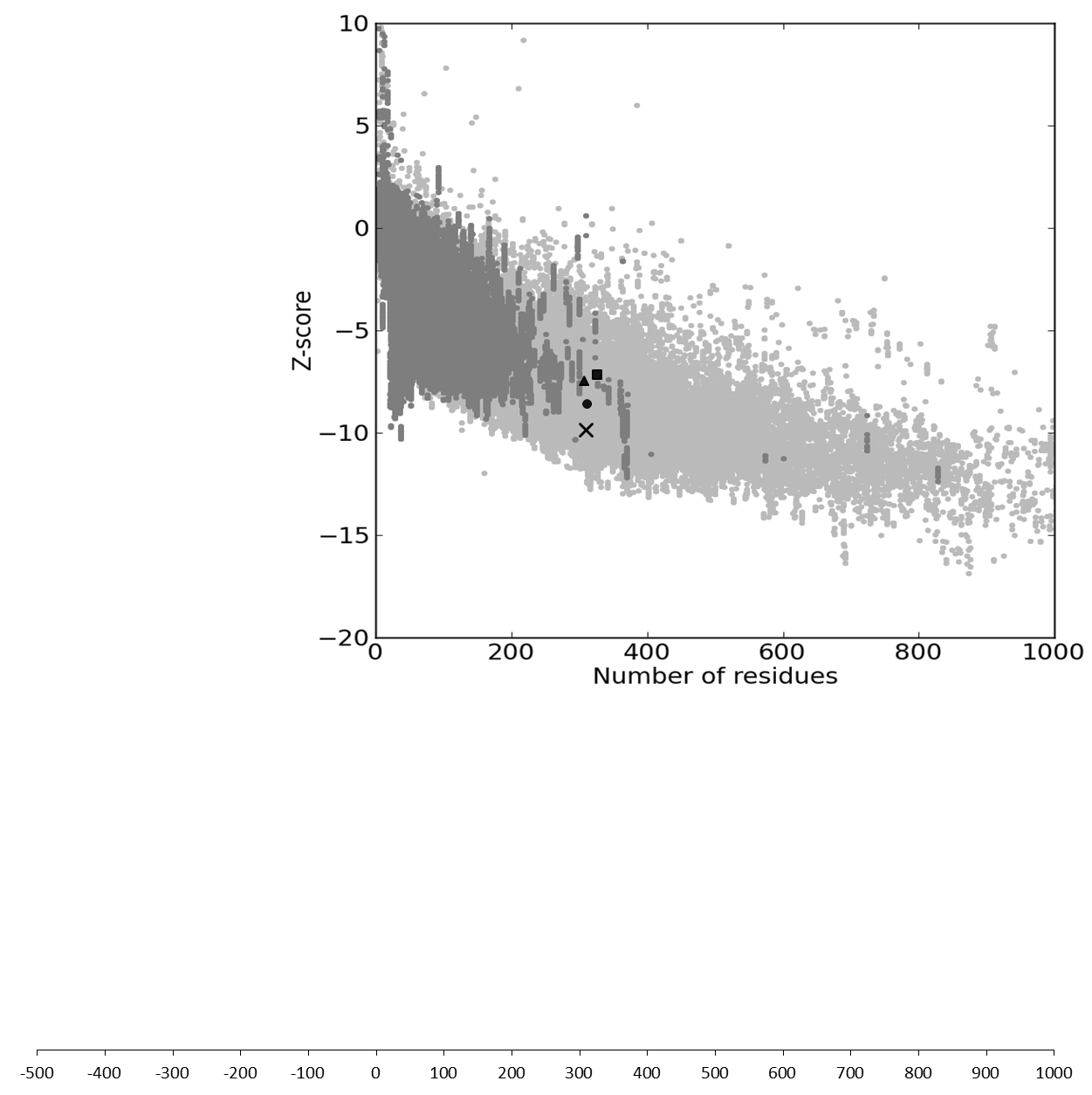
**

**Figure S6**

**Supplementary tables**

**Table S1.** The list of simulations for each enzyme with the temperatures given in both °C and K.

| **Enzyme** | **Simulated** **Temperature (ºC)** | **Simulated**  **Temperature (K)** | **Optimal**  **Temperature ( ºC)** |
| --- | --- | --- | --- |
| Est2 | 30  50  70  80  90 | 303  323  343  353  363 | 65 |
| EstB | 15  30  40  50  60 | 288  303  313  323  333 | 30 – 37 |
| EstP | 15  30  35  50 | 288  303  308  323 | 15 – 28 |
| EstS | 15  30  50 | 288  303  323 | 0 – 42 |

**Table S2.** Bacterial strains, plasmids and oligonucleotides used in this work.

| **Name** | **Description** | **Reference/ source** |
| --- | --- | --- |
| Strain | | |
| *E. coli* BL21 (DE3) | F^-^ *ompT hsdS*_B_ (r_B_^-^ m_B_^-^ ) *gal dcm* (DE3) | Novagen, Darmstadt, Germany |
| *E. coli* DH5α | *supE*44 Δ*lacU*169 (Ф80 lac*Z* ΔM15) *hsdR*17 *recA*1 *endA*1 *gyrA*96 *thi*-1 *relA*1 | Invitrogen, Karlsruhe, Germany |
| *A. acidocaldarius subsp.*  *acidocaldarius* | Wild type (DSMZ 446) | 1 |
| *Shewanella halifaxensis*  *HAW-EB4* | Wild type (DSMZ 17350) | 2 |
| *Burkholderia thailandensis*  *ATCC 700388* | Wild type (DSMZ 13276) | 3 |
| Plasmid | | |
| pET28a | Expression vector, *lacI*, kan^R^, T7-promotor, N-terminal His_6_-tag coding sequence (5369 bp) | Novagen, Darmstadt, Germany |
| pET28a-*EstBH6* | 986 bp fragment of *estB* cloned into *Nde*I and *Xho*I restriction sites of pET28a | This work |
| pET28a-*EstPH6* | 942 bp fragment of *estP* cloned into *Nde*I and *Xho*I restriction sites of pET28a | This work |
| pET28a-*EstSH6* | 941 bp fragment of *estS* cloned into *Nde*I and *Xho*I restriction sites of pET28a | This work |
| pET28a-*Est2H6* | 933 bp fragment of *est2* cloned into *Nde*I and *Xho*I restriction sites of pET28a | This work |
| Oligonuclotide | | |
| EstS-Up | GAAAGCCATATGCCGTTAGATCCAAAAGTCGCAC | *Nde*I |
| EstS-Down | GGAGCTCTCACTCGAGGATCTCTAACAGATCTTGCTT | *Xho*I |
| EstB-Up | CGATATCATATGTTTCGCCGTTTGCCCTTGACGATGCCG | *Nde*I |
| EstB-Down | GGAGCTCTCACTCGAGAGCGCCGTCGAATGCCGC | *Xho*I |
| Est2-Up | GAAAGCCATATGCCGCTCGATCCCGTCATTCAGC | *Nde*I |
| Est2-Down | ACGCGAGCTCCTAGGCCAGCGCGTCTCGAAGTTTCT | *Sac*I |

1. Darland, G. and T.D. Brpck, Bacillus acidocaldarius *sp.nov., an Acidophilic Thermophilic Spore forming Bacterium.* Journal of General Microbiology, 1971. **67**(1): p. 9-15.

2. Zhao, J.S., et al., Shewanella halifaxensis *sp. nov., a novel obligately respiratory and denitrifying psychrophile.* Int J Syst Evol Microbiol, 2006. **56**(Pt 1): p. 205-12.

3. Brett, P.J., D. DeShazer, and D.E. Woods, Burkholderia thailandensis *sp. nov., a* Burkholderia pseudomallei*-like species.* Int J Syst Bacteriol, 1998. **48 Pt 1**: p. 317-20.

**Bezeichnung DNA-Sequenz (in 5’→ 3’ Richtung)**

**Table S3:** Lipases with known structure and number of homologous enzymes.

| **Template enzyme** | | | **Number of homologs*** | | | |
| --- | --- | --- | --- | --- | --- | --- |
| **PDB ID** | **Function** | **Bacterium** | **psychroph.** | **psychrot.** | **mesoph.** | **thermoph.** |
| 1EVQ | esterase | *Alicyclobacillus acidocaldarius* | **2** | **4** | **3** | **4** |
| 1T4M | esterase | *Bacillus amyloliquefaciens* | **5** | **1** | **-** | **3** |
| 2ES4 | lipase | *Burkholderia glumae* | **6** | **2** | **-** | **1** |
| 1JKM | brefeldin A esterase | *Bacillus subtilis* | **4** | **-** | **-** | **4** |
| 1YS2 | lipase | *Burkholderia cepacia* | **6** | **1** | **-** | **--** |
| 3E0X | lipase, esterase | *Clostridium acetobutylicum* | **5** | **--** | **-** | **5** |
| 1YZF | lipase, acylhydrolase | *Enterococcus faecalis* | **8** | **--** | **-** | **3** |
| 3I6Y | esterase | *Oleispira antarctica* | **-** | **2** | **--** | **2** |
| 3D3N | lipase, esterase | *Lactobacillus plantarum* | **-** | **--** | **--** | **3** |
| 2ORY | lipase | *Photobacterium sp. M37* | **-** | **--** | **--** | **1** |
| 2Z8Z | lipase | *Pseudomonas sp. MIS38* | **3** | **--** | **-** | **-** |
| 1EX9 | lipase | *Pseudomonas aeruginosa* | **--** | **--** | **-** | **6** |
| 2FX5 | lipase | *Pseudomonas mendocina* | **--** | **--** | **-** | **3** |
| 2QUB | lipase | *Serratia marcescens* | **1** | **--** | **-** | **--** |
| 2PBL | thioesterase | *Silicibacter sp. TM1040* | **2** | **--** | **-** | **3** |
| 1JI3 | lipase | *Geobacillus stearothermophilus* | **--** | **2** | **--** | **-** |
| 1JFR | lipase | *Streptomyces exfoliatus* | **2** | **--** | **--** | **-** |

* Black shading indicate habitat of template enzyme, grey shading indicates habitat with existing homolog and white fields indicate habitats without identified homologs.

**Table S4:** Enzymes homologous to Est2

| **Source organism** | **Habitat** | **Homologous enzymes** | | | |
| --- | --- | --- | --- | --- | --- |
|  |  | **UniProt ID** | **Function** | **No. of residues** | **Sequence identity (%)** |
| *Bacillus megaterium* DSM319 | mesophile | D5DEE6 | Esterase | 310 | 50 |
| *Brevibacillus brevis* NBRC 100599 | mesophile | C0ZCZ0 | Putative Lipase/Esterase | 312 | 48 |
| ***Burkholderia thailandensis* E264** | **mesophile** | **Q2SWM9** | **Esterase** | **327** | **45** |
| *Rodoferax ferrireducens* T118 | pschrotroph | Q21VV3 | Alpha/beta hydrolase fold-3domain protein | 327 | 38 |
| *Shewanella pealeana* ATCC 700345 | pschrotroph | A8H9V4 | Alpha/beta hydrolase domain protein | 312 | 38 |
| *Shewanella piezotolerans* WP3 | pschrotroph | B8CV73 | Esterase/Lipase | 313 | 37 |
| ***Pseudomonas sp*. B11-1** | **pschrotroph** | **O52270** | **Esterase** | **308** | **47** |
| *Shewanella loihica* PV-4 | psychrophile | A3QGD9 | Alpha/beta hydrolase fold-3 domain protein | 320 | 40 |
| ***Shewanella halifaxensis* HAW –EB4** | **psychrophile** | **B0TNQ0** | **Alpha/beta hydrolase fold-3 domain protein** | **312** | **38** |

**Table S5:** Amino acid composition comparison.

| **Amino**  **acid** | **mol%*** | | | | **R^2^** |
| --- | --- | --- | --- | --- | --- |
|  | **EST2** | **EstB** | **EstP** | **EstS** |  |
| Cys | 0.32 | 0.92 | 2.27 | 1.28 | 0.59 |
| Asn | 1.94 | 0.92 | 2.60 | 2.88 | 0.26 |
| Thr | 2.58 | 4.28 | 3.25 | 4.49 | 0.52 |
| Glu | 6.45 | 4.59 | 5.84 | 5.45 | 0.20 |
| Pro | 9.03 | 6.42 | 6.17 | 6.41 | 0.83 |
| Val | 7.42 | 6.42 | 5.52 | 5.13 | 0.98 |
| Ala | 11.94 | 17.13 | 14.94 | 9.94 | 0.01 |
| Arg | 5.16 | 7.65 | 6.49 | 4.49 | 0.00 |
| Asp | 6.77 | 7.65 | 7.14 | 7.37 | 0.35 |
| Gln | 3.87 | 2.45 | 5.84 | 4.81 | 0.23 |
| Gly | 6.77 | 7.65 | 6.49 | 6.73 | 0.03 |
| His | 2.58 | 3.67 | 1.95 | 2.88 | 0.01 |
| Ile | 3.23 | 2.75 | 2.27 | 6.09 | 0.17 |
| Leu | 11.29 | 9.79 | 12.66 | 10.90 | 0.02 |
| Lys | 3.87 | 2.14 | 0.65 | 3.85 | 0.12 |
| Met | 1.94 | 2.14 | 2.27 | 1.92 | 0.08 |
| Phe | 3.87 | 4.59 | 4.87 | 3.21 | 0.00 |
| Ser | 4.52 | 3.98 | 4.55 | 5.45 | 0.26 |
| Trp | 1.29 | 1.22 | 0.97 | 1.28 | 0.15 |
| Tyr | 5.16 | 3.67 | 3.25 | 5.45 | 0.04 |

*Number of amino acids and molecular weights of studied proteins used to calculate molar ratio for each amino acid were Est2: 310aa, 34303Da; EstB: 327aa, 35500Da; EstP: 308aa, 33711Da; EstS: 312aa, 34781Da

**Table S6:** Thermostability of selected homologous enzymes

|  | **min**  **°C** | **0** | **20** | **40** | **60** | **80** | **100** | **120** | **140** | **160** | **180** |
| --- | --- | --- | --- | --- | --- | --- | --- | --- | --- | --- | --- |
| **EstB** | **5** | 100 | 85 | 84 | 86 | 83 | 81 | 82 | 80 | 85 | 81 |
|  | **10** | 100 | 96 | 94 | 93 | 93 | 93 | 95 | 96 | 96 | 93 |
|  | **15** | 100 | 100 | 101 | 100 | 102 | 103 | 101 | 103 | 103 | 101 |
|  | **20** | 100 | 83 | 83 | 81 | 81 | 79 | 78 | 78 | 78 | 77 |
|  | **25** | 100 | 78 | 74 | 73 | 71 | 70 | 69 | 69 | 67 | 68 |
|  | **30** | 100 | 73 | 69 | 62 | 58 | 53 | 47 | 41 | 37 | 32 |
|  | **35** | 100 | 67 | 61 | 53 | 46 | 41 | 33 | 25 | 18 | 12 |
|  | **40** | 100 | 51 | 45 | 38 | 28 | 24 | 21 | 20 | 9 | 5 |
|  | **45** | 100 | 39 | 33 | 28 | 22 | 19 | 12 | 6 | 1 | 1 |
|  | **50** | 100 | 11 | 5 | 3 | 2 | 1 | 0 | 0 | 0 | 1 |
|  | **55** | 100 | 3 | 1 | 1 | 0 | 0 | 0 | 0 | 0 | 0 |
|  | **60** | 100 | 0 | 0 | 0 | 0 | 0 | 0 | 0 | 0 | 1 |
|  | **65** | 100 | 0 | 0 | 0 | 0 | 0 | -1 | 0 | 0 | 0 |
|  | **70** | 100 | 0 | 0 | 1 | 0 | 0 | 0 | 0 | 0 | -1 |
|  | **75** | 100 | 0 | 0 | 0 | 0 | 0 | 0 | 0 | 0 | 0 |
|  | **80** | 100 | 0 | 0 | 0 | 0 | 0 | 0 | 0 | 0 | 1 |
| **EstP** | **5** | 100 | 68 | 67 | 67 | 68 | 68 | 67 | 68 | 67 | 68 |
|  | **10** | 100 | 91 | 93 | 93 | 94 | 93 | 94 | 94 | 94 | 93 |
|  | **15** | 100 | 96 | 97 | 97 | 98 | 97 | 97 | 97 | 98 | 97 |
|  | **20** | 100 | 102 | 101 | 100 | 100 | 101 | 100 | 101 | 100 | 100 |
|  | **25** | 100 | 79 | 79 | 77 | 78 | 78 | 79 | 79 | 79 | 79 |
|  | **30** | 100 | 58 | 58 | 58 | 57 | 58 | 57 | 57 | 57 | 57 |
|  | **35** | 100 | 35 | 34 | 32 | 32 | 32 | 33 | 32 | 32 | 32 |
|  | **40** | 100 | 27 | 27 | 25 | 24 | 22 | 20 | 19 | 18 | 17 |
|  | **45** | 100 | 8 | 5 | 4 | 0 | 0 | 0 | 1 | 1 | 1 |
|  | **50** | 100 | 5 | 0 | 0 | 0 | 1 | 1 | 0 | 0 | -1 |
|  | **55** | 100 | 1 | 1 | 0 | 1 | 1 | 0 | 0 | 1 | 0 |
|  | **60** | 100 | 0 | 0 | 1 | 0 | 0 | 0 | 0 | 0 | 1 |
|  | **65** | 100 | 0 | 0 | 1 | 1 | 0 | 0 | 0 | 0 | 1 |
|  | **70** | 100 | 0 | 0 | 0 | 0 | 0 | 0 | 0 | 0 | 0 |
|  | **75** | 100 | 0 | 0 | 0 | 0 | 1 | 0 | 1 | 0 | 0 |
|  | **80** | 100 | 0 | 0 | 0 | 0 | 0 | 0 | 0 | 0 | 1 |
| **EstS** | **5** | 100 | 101 | 101 | 95 | 96 | 97 | 95 | 95 | 92 | 97 |
|  | **10** | 100 | 100 | 100 | 100 | 100 | 100 | 100 | 100 | 100 | 99 |
|  | **15** | 100 | 99 | 99 | 99 | 99 | 99 | 99 | 99 | 99 | 99 |
|  | **20** | 100 | 102 | 100 | 100 | 102 | 103 | 103 | 100 | 102 | 101 |
|  | **25** | 100 | 102 | 91 | 87 | 82 | 81 | 82 | 81 | 81 | 80 |
|  | **30** | 100 | 54 | 25 | 17 | 12 | 9 | 8 | 6 | 6 | 6 |
|  | **35** | 100 | 31 | 3 | 1 | 1 | 0 | 0 | 0 | 0 | 0 |
|  | **40** | 100 | 3 | 0 | 0 | 0 | 0 | 0 | 0 | 0 | 0 |
|  | **45** | 100 | 0 | 0 | 0 | 0 | 0 | 0 | 0 | 0 | 0 |
|  | **50** | 100 | 0 | 0 | 0 | -1 | 0 | 0 | 0 | 0 | 0 |
|  | **55** | 100 | 0 | 0 | 0 | 0 | 0 | -1 | -1 | 0 | -1 |
|  | **60** | 100 | 0 | 0 | 0 | 0 | 0 | 0 | 0 | 0 | 0 |
|  | **65** | 100 | 0 | 0 | 0 | 0 | 0 | -1 | 0 | 0 | 0 |
|  | **70** | 100 | 0 | 0 | 1 | 0 | 0 | 0 | 0 | 0 | -1 |
|  | **75** | 100 | 0 | 0 | 0 | 0 | 0 | 0 | 0 | 0 | 1 |
|  | **80** | 100 | 0 | 0 | 0 | 0 | 0 | 0 | 0 | 0 | 1 |
| **Est2** | **5** | 100 | 55 | 53 | 53 | 54 | 51 | 49 | 52 | 55 | 55 |
|  | **10** | 100 | 61 | 61 | 62 | 57 | 56 | 54 | 53 | 50 | 52 |
|  | **15** | 100 | 68 | 67 | 67 | 68 | 66 | 60 | 61 | 62 | 58 |
|  | **20** | 100 | 84 | 83 | 68 | 66 | 67 | 60 | 59 | 61 | 60 |
|  | **25** | 100 | 81 | 79 | 75 | 75 | 77 | 74 | 69 | 69 | 68 |
|  | **30** | 100 | 83 | 80 | 80 | 76 | 76 | 75 | 76 | 75 | 72 |
|  | **35** | 100 | 87 | 85 | 83 | 84 | 80 | 75 | 75 | 74 | 74 |
|  | **40** | 100 | 86 | 84 | 84 | 80 | 82 | 81 | 81 | 81 | 80 |
|  | **45** | 100 | 86 | 85 | 86 | 86 | 85 | 80 | 81 | 78 | 79 |
|  | **50** | 100 | 88 | 88 | 89 | 85 | 87 | 85 | 83 | 81 | 79 |
|  | **55** | 100 | 94 | 96 | 92 | 92 | 91 | 90 | 89 | 87 | 86 |
|  | **60** | 100 | 97 | 95 | 96 | 90 | 93 | 87 | 89 | 89 | 88 |
|  | **65** | 100 | 99 | 96 | 99 | 92 | 99 | 91 | 91 | 95 | 97 |
|  | **70** | 100 | 104 | 107 | 107 | 101 | 103 | 103 | 97 | 97 | 97 |
|  | **75** | 100 | 96 | 93 | 92 | 92 | 89 | 88 | 88 | 84 | 85 |
|  | **80** | 100 | 77 | 60 | 57 | 48 | 41 | 30 | 19 | 16 | 9 |

**Table S7:** Stereo-chemical quality by PROCHECK and structure validation using PROSA and QMEAN.

| **Enzyme** | **PROCHECK analysis** | | | | **PROSA**  **Z-score** | **Q-MEAN**  **Z-score** |
| --- | --- | --- | --- | --- | --- | --- |
|  | **Core (%)** | **Allowed (%)** | **Generously (%)** | **Disallowed (%)** |  |  |
| EstS | 87,4 | 9,7 | 0,7 | 2,2 | -8,63 | 0,76 |
| EstB | 89,6 | 7,2 | 1,1 | 2,2 | -7,19 | 0,77 |
| EstP | 90,6 | 6,7 | 1,9 | 0,7 | -7,54 | 0,82 |
| Est2 | 86,4 | 12,1 | 0,8 | 0,8 | -9,88 | 0,81 |

| **Table S8:** Structural conservation of ion bridges.   | *To ion bridges detected in Est2 were assigned numbers (Nr.) in order to correlate each ion bridge with the respective homologous structure. Numbers (Nr.) given for EstS, EstB and EstP correspond to the ion bridges of Est2. Grey shading indicates four ion bridges conserved among all four structures. |
| --- | --- |
